# Analysis of Indicators for Assessing the Risk of Progression from PACS to APAC and the Degree of Intraocular Pressure Elevation in APAC Using AS-OCT

**DOI:** 10.1155/2024/8368315

**Published:** 2024-09-06

**Authors:** Rumin Zhao, Qiaofang Du, Yunlong Wu, Wenhui Geng, Zijian Zhang, Bojun Zhao

**Affiliations:** ^1^ Shandong University of Traditional Chinese Medicine, Jinan, Shandong, China; ^2^ Shandong Lunan Eye Hospital, Linyi, China; ^3^ Department of Ophthalmology Shandong Provincial Hospital, Jinan, Shandong, China

## Abstract

**Objective:**

The aim of this study is to quantify anterior chamber parameters to provide potential risk indicators for evaluating the progression of primary angle-closure suspect (PACS) eyes to acute primary angle closure (APAC) and the degree of intraocular pressure elevation in patients with APAC utilizing anterior segment optical coherence tomography (AS-OCT).

**Methods:**

Tomey CASIA2 AS-OCT was used to quantitatively measure various anterior chamber parameters, including anterior chamber depth (ACD), anterior chamber volume (ACV), lens thickness (LT), lens vault (LV), iris-trabecular contact index (ITC), iris thickness (IT), iris volume (IV), iris curvature (IC), iris area (IA), and iris thickness (IT), in APAC eyes (30 eyes) and contralateral PACS eyes (30 eyes) of 30 patients. The differences in these parameters between the two groups and their relationship with intraocular pressure were analyzed.

**Results:**

Compared to the PACS group, the APAC group exhibited significantly smaller IA and IC, and significantly larger pupil diameter (PD) and ITC (*P* < 0.05). There were no statistically significant differences in ACV, ACD, ACW, ACA, LV, IV, and IT750/2000 between the two groups. In APAC eyes, multivariable linear regression analysis showed a significant negative correlation between intraocular pressure and IV (*β* = −1.85; 95% confidence interval: −2.77 to −0.93; *P*=0.001), while no correlation was found in PACS eyes. In all 60 eyes, LT showed a negative correlation with ACV, ACD, ACA, and nasal IT750, and a positive correlation with LV and nasal IC.

**Conclusion:**

AS-OCT has multiple advantages in evaluating various anterior chamber parameters in patients with glaucoma. IA may serve as a predictive indicator of the progression of eyes from PAC or APAC. A significant negative correlation was found between intraocular pressure and IV during APAC attacks. LT can be considered a predictive factor for the occurrence of primary angle-closure disease.

## 1. Introduction

Glaucoma is the leading cause of irreversible blindness worldwide. Primary angle-closure glaucoma (PACG) accounts for 25% of all types of glaucoma globally. It is the most common type of glaucoma in the Chinese population [1]. Acute primary angle closure (APAC), classified in the International Society of Geographical and Epidemiological Ophthalmology (ISGEO) staging system, is a special form of primary angle-closure disease (PACD) and represents an ophthalmic emergency, characterized by the sudden closure of the anterior chamber angle leading to a rapid increase in intraocular pressure (IOP) [2]. Without prompt treatment, patients may experience optic nerve damage and progressive vision loss [3]. If the contralateral eye exhibits a shallow anterior chamber, narrow angle, and short axial length, even without elevated IOP, it is considered as a primary angle-closure suspect (PACS) in the ISGEO staging system [2].

In clinical practice, both ultrasound biomicroscopy (UBM) and anterior segment optical coherence tomography (AS-OCT) can be used for quantitative measurement of anterior chamber parameters [4]. Compared with UBM, AS-OCT has the following advantages: noncontact, time-saving, and does not require a supine position during examination. Therefore, AS-OCT is widely used to obtain high-resolution anterior chamber images [5]. It has gone through several generations of innovation, from time-domain OCT (TD-OCT), frequency-domain OCT (SD-OCT), to swept frequency OCT (SSOCT). CASIA2 SS-AS-OCT has a high scanning speed, providing higher depth sensitivity and higher resolution images. This makes it possible to obtain image parameter data from the cornea to the lens in just a few seconds.

In patients with PACD, if one eye presents with APAC, the contralateral eye often exhibits PACS. Therefore, it is important to assess the risk of the contralateral eye transitioning from PACS to APAC. This study aims to compare APAC eyes and PACS eyes in the same patient, utilizing AS-OCT quantitative measurements of anterior chamber parameters, to provide potential risk indicators for evaluating the progression of PACS to APAC in patients with PACS and the degree of IOP elevation when APAC attacks.

## 2. Research Methods

### 2.1. Study Population

This study is a retrospective study conducted in accordance with the principles of the Helsinki Declaration and approved by the Ethics Committee of Shandong Lunan Eye Hospital. The analyzed data were collected from patients with APAC who received continuous treatment in the glaucoma department of Shandong Lunan Eye Hospital from November 2022 to May 2023. A total of 30 patients (60 eyes) were included in the study, comprising 30 APAC eyes and 30 contralateral PACS eyes. The diagnosis of APAC and PACS was based on the diagnostic criteria of the International Society of Geographical and Epidemiological Ophthalmology (ISGEO) [6]. All study participants were over 60 years old and had mild cataracts. The exclusion criteria were as follows: (1) patients with simultaneous acute attacks in both the eyes; (2) patients with a history of recurrent minor attacks and obvious optic nerve damage; (3) patients who had previously undergone any laser or intraocular surgery (including LPI, laser iridoplasty, trabeculectomy, and other glaucoma-related treatments, as well as any other intraocular surgery); (4) patients with known eye diseases that affect the anterior chamber anatomy, such as ciliary or iris cysts, a history of trauma, and the use of medications that affect iris configuration; (5) patients with a history of any other intraocular diseases or concurrent intraocular diseases, such as uveitis, diabetic retinopathy, macular degeneration, or retinitis pigmentosa; and (6) patients with life-threatening or systemic diseases that may affect the study results.

### 2.2. Ophthalmic Examinations

All patients underwent baseline ophthalmic examinations at their initial visit, including visual acuity (logMAR), intraocular pressure (IOP, iCare tonometry), slit-lamp biomicroscopy, ocular ultrasound, undilated fundus examination, anterior chamber angle examination, UBM, and anterior segment optical coherence tomography (AS-OCT) examination.

### 2.3. AS-OCT Image Acquisition and Parameter Measurements

AS-OCT imaging was obtained before the initiation of acute attack treatment by the same experienced ophthalmic technician. All patients underwent AS-OCT examination in a dark room environment using the Tomey CASIA2 device (Tomey Corporation, Nagoya, Japan). CASIA2 utilized a 1310-nm swept-source laser wavelength with a frequency of 0.3 s. Continuous scanning was performed in the “anterior chamber angle” mode. Images were obtained and analyzed using the provided software by the manufacturer. The anterior segment structures were automatically analyzed by the built-in software and the measurement results were provided after marking the scleral spur (SS). SS was defined as the point of curvature change at the junction of the cornea and sclera, where the sclera protrudes inward. A trained technician marked the SS without knowledge of the anterior chamber angle grading. The analyzed parameters included iris-trabecular contact index (ITCI, defined as the percentage of iris-trabecular contact area to the total measured area), anterior chamber volume (ACV), anterior chamber depth (ACD), anterior chamber width (ACW), anterior chamber area (ACA), lens vault (LV, defined as the distance between the intersection of the vertical bisector of SS connection and lens and the midpoint of SS connection), lens thickness (LT), iris area (IA), iris volume (IV), iris curvature (IC), and iris thickness at 750/2000 *μ*m (IT750/2000).

### 2.4. Management of Acute Angle-Closure Glaucoma Attacks

A standard protocol was followed for the management of acute angle-closure glaucoma (AAC) attacks. Initially, local and systemic medications were used to alleviate the attack. If the symptoms did not subside after 2 hours of medication, laser peripheral iridoplasty (LPIP, energy: 300–340 mW, spot size: 500 *μ*m, exposure time: 700 ms) was performed. If the symptoms still persisted and corneal edema was severe, leading to the lens extraction surgery being unfeasible, a low-dose transscleral cyclophotocoagulation (TSCPC) was further conducted to reduce IOP. All patients underwent ultrasound phacoemulsification combined with gonioscopy-assisted goniosynechialysis, under good corneal transparency and controlled intraocular inflammatory. The surgeries were performed by an experienced glaucoma specialist under topical anesthesia.

### 2.5. Statistical Description

Statistical analysis was performed using the Free Statistics software version 1.7.1. Categorical variables were described as frequencies (%), while continuous variables were described as mean ± standard deviation (SD) or median (range), if the data were not normally distributed. The paired *t*-test was applied for the parameters within APAC and PACS groups in Tables 1 and 2. Multivariable linear regression models were employed to explore correlations between the various parameters. A *P* value less than 0.05 was considered statistically significant.

## 3. Results

### 3.1. Description of Basic Characteristics of the Study Population

The average age of the 30 patients included in the study was 66.6 ± 5.8 years old. Among them, 80% were females. Four patients (13.3%) had concomitant diabetes, and eight patients (26.7%) had concomitant hypertension. The average BMI was 24.7 ± 3.2 kg/m^2^. The mean initial IOP in the APAC eyes was 42.5 ± 16.2 mmHg, and the mean IOP after pressure control was 19.0 ± 7.5 mmHg. The mean IOP in the PACS eyes was 15.6 ± 9.8 mmHg. The initial visual acuity in the APAC eyes was 1.7 ± 1.1, and the preoperative visual acuity was 1.0 ± 0.6. In the PACS eyes, the visual acuity was 0.3 ± 0.2. The mean axial length was 22.0 ± 0.7 mm in both the APAC and PACS groups. The mean lens thickness was 5.1 ± 0.3 mm in both the groups. The mean corneal horizontal diameter (WTW) was 11.2 ± 0.4 mm in the APAC group and 11.2 ± 0.6 mm in the PACS group. The mean anterior chamber depth (ACD) was 1.7 ± 0.3 mm in the APAC group and 1.8 ± 0.3 mm in the PACS group.  Table 1 summarizes the various preoperative variables.

### 3.2. Comparison of Preoperative Parameters between the APAC and PACS Groups

Compared to the PACS group, the APAC group showed significantly smaller IA and IC, as well as larger pupil diameter (PD) and ITCI (all *P* < 0.05). There were no statistically significant differences between the two groups in terms of ACV, ACD, ACW, ACA, LV, IV, and iris thickness at 750/2000 *μ*m (IT750/2000) (all *P* > 0.05). Please refer to Table 2 for details.

### 3.3. Correlation between IOP and Iris Volume

In APAC eyes during acute attacks, multivariable linear regression analysis showed a significant negative correlation between IOP and IV.  Table 3 shows the coefficient and 95% confidence interval (CI) for the three models. In model II, for each 1 mmHg increase in IOP, IV decreased by 1.85 mm^3^ after adjustment for age, sex, body mass index (BMI), hypertension, and diabetes (*β* = −1.85, 95% CI: −2.77 to −0.93, *P*=0.001). On the other hand, no correlation was observed in PACS eyes for the three models (nonadjusted *β* = 0, 95% CI: −0.15 to 0.15, *P*=0.995; model I *β* = −0.02, 95% CI: −0.19 to 0.15, *P*=0.839; model II *β* = −0.02, 95% CI: −0.20 to 0.15, *P*=0.801) (see Table 4).

### 3.4. Correlation between Lens Thickness (LT) and Anterior Chamber and Iris Parameters

**Figure 1:**
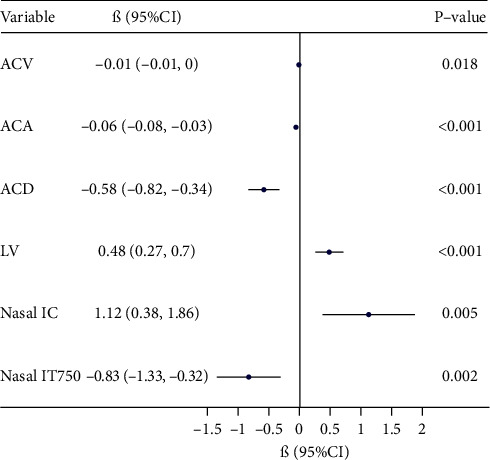
Linear regression analysis of the correlation between lens thickness (LT) and various indicators in all 60 eyes (adjusted for age, BMI, gender, diagnosis, hypertension, and diabetes).

**Figure 2:**
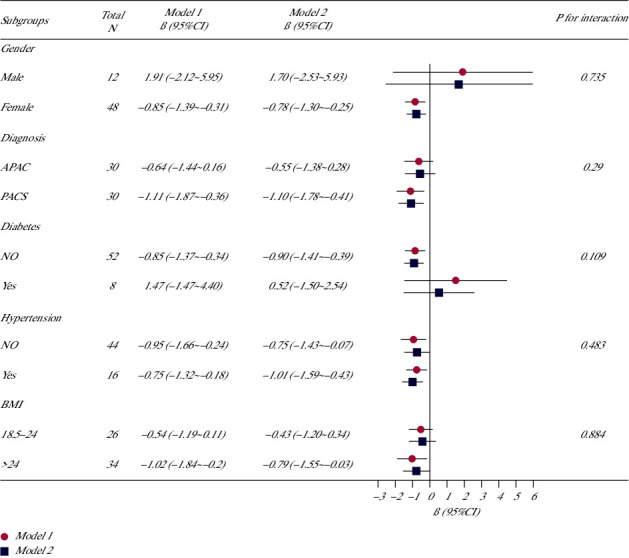
Association between lens thickness (LT) and nasal iris thickness at 750 *μ*m (IT750) according to baseline characteristics. Model I: adjusted for age, BMI, and gender. Model II: adjusted for the variables in model I plus diagnosis, hypertension, and diabetes.

In all 60 eyes, LT showed a negative correlation with ACV (*β* = −0.01, 95% CI: −0.01 to 0, *P*=0.018), ACD (*β* = −0.58, 95% CI: −0.82 to −0.34, *P*=0.001), ACA (*β* = −0.06, 95% CI: −0.08 to −0.03, *P* < 0.001), and nasal IT750 (*β* = −0.83, 95% CI: −1.33 to −0.32, *P*=0.002) and a positive correlation with LV (*β* = 0.48, 95% CI: 0.27 to 0.70, *P*=0.001) and nasal IC (*β* = 1.12, 95% CI: 0.38 to 1.86, *P*=0.005), using multivariable linear regression analysis after adjustment for age, sex, BMI, diagnosis, hypertension, and diabetes (see Table 5 and Figure 1). Among the abovementioned parameters, only the relationship between LT and IT750 has not been reported previously; hence, we further adopted stratified analysis to assess whether the correlation of LT with IT750 was robust in different subgroups (Figure 2). The subgroup analyses were performed using different stratified logistic regression models according to sex, diagnosis, BMI, hypertension, and diabetes. No significant interaction was observed in all five subgroups (all *P* > 0.05).

## 4. Discussion

Over the years, known risk factors for APAC, such as shallow anterior chamber, narrow peripheral angles, thicker lens, anterior lens position, and shorter axial length, have been identified using ultrasound biomicroscopy, gonioscopy, and A-scan biometry. Although these traditional tools have helped us understand the basic pathogenesis of APAC, they have limitations such as being invasive, nonautomated, subjective, time-consuming, and nonquantitative. AS-OCT has gained popularity due to its advantages of upright positioning, noncontact measurement, rapidity, semiautomation, objectivity, high resolution, quantification, and high repeatability [7, 8]. In this study, Tomey CASIA2 AS-OCT was used to assess the differences in anterior segment parameters between APAC and PACS within Chinese patients. Compared to previous AS-OCT devices, CASIA2 offers advantages such as faster scanning speed and built-in programs, allowing for the measurement of average anterior chamber parameters in 360° within seconds. It is widely utilized for anterior segment evaluation in patients with PAC disease [9].

This study found that, compared to the PACS group, the APAC group had larger pupil diameter (PD) and smaller iris area (IA) and iris curvature (IC). This finding aligns with previous research that discovered an interesting phenomenon: for each 1 mm increase in PD in PACG eyes, there is a loss of 0.145 mm^2^ in IA in the same eye and a loss of 0.161 mm^2^ in the fellow eye. In normal control eyes, the loss is 0.165 mm^2^ [10]. In other words, in APAC eyes, due to the dilation of the pupil, there is a corresponding loss in IA, which is consistent with the findings of our study. Combining previous research with our results indicates that when comparing PACS eyes to PACG eyes with the same increase in PD diameter, PACG eyes have a smaller loss in IA. This suggests that in clinical practice, during AS-OCT examination under darkroom conditions for patients with PACS without a history of acute attacks in both eyes, a larger IA may indicate a smaller loss in IA, making the eye more prone to developing PAC or PACG. Therefore, IA could potentially serve as a predictive indicator for the progression from PACS to PAC or PACG. In addition, our study also found a negative correlation between IOP and iris volume (IV) during acute attacks in APAC eyes, while there was no correlation between IOP and IV in the PACS group. This phenomenon was explainable. IV may be reduced as IOP increases as a result of IOP-related iris compression or anterior segment ischemia.

This study also found a decrease in IC in the APAC group compared to that in the PACS group. This could be attributed to the acute increase in IOP during an acute angle-closure attack, which leads to angle closure, obstruction of aqueous outflow, and increased pressure in both the anterior and posterior chambers, thereby resulting in a reduction in IC. However, Guzman et al. conducted a clinical-based study in Singapore, specifically focusing on patients with PACD of Chinese ethnicity, and found no significant differences in IA and IC between PACS and PAC/PACG eyes [11]. This discrepancy may be attributed to the differences in the study population. Our study primarily focused on APAC eyes, which represent a distinct subtype of PACG, characterized by a rapid increase in IOP and pupil dilation, thereby leading to changes in iris parameters. This discrepancy between our study and the study by Guzman et al. highlights the need for studies with larger sample sizes to further validate these findings.

Lens thickness (LT) is negatively correlated with anterior chamber volume (ACV), anterior chamber depth (ACD), and anterior chamber area (ACA), while it is positively correlated with lens vault (LV) and IC. Saxena et al. also found that an increase of 0.01 mm in LT was associated with an 11% increase in the occurrence of ACG [12], which aligns with our study's findings. A thicker lens implies a larger LV, and several population-based studies have demonstrated that a thicker and more anteriorly positioned lens can predict angle closure [13]. LT has been reported as a predictive factor for PACD, with a larger LT indicating a greater curvature of the anterior surface of the lens with aging, thereby increasing the likelihood of pupillary block, which is considered a key element in PACD [14, 15]. This also confirms the role of the lens in anterior chamber crowding and pupillary block. Some studies have reported that a larger iris curvature is associated with a greater increase in anterior chamber angle width after LPI, which can be attributed to the relief of the pupillary block and subsequent widening of the angle. This provides further evidence that when LT is larger, IC is larger, and the pupillary block is more pronounced, thus resulting in a shallower anterior chamber. Conversely, when LT is smaller, IC is smaller, indicating a flatter iris, and the anterior chamber is deeper [16, 17].

However, this study also has certain limitations. First, the sample size was small, and further research with a larger number of participants is needed. Second, although we usually perform AS-OCT at the time of patient admission, some patients may have already been treated with IOP-lowering drugs in other hospitals, which may affect certain parameters. Lastly, this study primarily focused on the preoperative parameters of the two groups, and it would be beneficial to supplement this research by examining parameter changes at various postoperative time points through regular follow-ups.

## 5. Conclusions

AS-OCT offers several advantages in assessing various parameters of the anterior segment in patients with glaucoma. IA may serve as a predictive indicator for the progression from PACS to PAC or APAC. A significant negative correlation was found between IOP and IV during APAC attacks. LT can be considered a predictive factor for the occurrence of PACD, in which pupillary block is a key contributing factor.

## Figures and Tables

**Table 1:** Demographics and characteristics of participants.

Variable		APAC (*n* = 30)	PACS (*n* = 30)	*P* value
VA-first visit (logMAR)	≤0.1	0 (0)	3 (10)	—
	>0.1, ≤0.3	0 (0)	11 (36.7)	—
	>0.3, ≤1.0	9 (30)	15 (50)	—
	>1.0	21 (70)	1 (3.3)	—

IOP-first visit (mmHg)		42.5 ± 16.2	15.6 ± 9.8	<0.001

IOP-preoperation (mmHg)		19.0 ± 7.5	14.4 ± 2.7	0.002

Measures to reduce IOP	Medication	14 (46.7)	—	—
	Medication + LPIP	9 (30)	—	—
	Medication + LPIP + low-dose TSCPC	7 (23.3)	—	—

AL (mm)		22.0 ± 0.7	22.0 ± 0.7	0.888

LT (mm)		5.1 ± 0.3	5.1 ± 0.3	0.856

WTW (mm)		11.2 ± 0.4	11.2 ± 0.6	0.943

ACD (mm)		1.7 ± 0.3	1.8 ± 0.3	0.052

**Table 2:** Comparison of AS-OCT parameters between the APAC group and the PACS group.

Variable	Total (*n* = 60)	APAC (*n* = 30)	PACS (*n* = 30)	*P* value
ACV (mm^3^)	66.166 ± 13.884	66.447 ± 12.763	65.885 ± 15.137	0.877
IV (mm^3^)	27.897 ± 6.188	26.464 ± 5.442	29.330 ± 6.636	0.073
ACD (mm)	1.735 ± 0.269	1.668 ± 0.269	1.802 ± 0.257	0.052
LV (mm)	0.829 ± 0.279	0.818 ± 0.324	0.841 ± 0.229	0.754
ACW (mm)	10.623 ± 0.531	10.536 ± 0.542	10.710 ± 0.515	0.207
ACA (mm^3^)	11.421 ± 2.692	11.511 ± 3.128	11.332 ± 2.224	0.800
PD (mm)	3.929 ± 1.298	4.853 ± 0.859	3.005 ± 0.961	<0.001
ITC	66.845 ± 31.594	90.397 ± 12.290	43.293 ± 27.058	<0.001
Mean IA (mm^2^)	1.300 ± 0.400	1.200 ± 0.300	1.300 ± 0.400	0.042
Mean IC	0.200 ± 0.100	0.100 ± 0.100	0.200 ± 0.100	0.004
Mean IT750	0.335 ± 0.109	0.350 ± 0.108	0.320 ± 0.111	0.294
Mean IT2000	0.344 ± 0.126	0.336 ± 0.112	0.352 ± 0.140	0.616
Nasal IT750 (mm)	0.348 ± 0.130	0.362 ± 0.138	0.333 ± 0.120	0.375
Nasal IT2000 (mm)	0.345 ± 0.159	0.347 ± 0.149	0.344 ± 0.171	0.932
Temporal IT750 (mm)	0.334 ± 0.129	0.338 ± 0.147	0.331 ± 0.112	0.837
Temporal IT2000 (mm)	0.318 ± 0.139	0.324 ± 0.114	0.312 ± 0.162	0.732

**Table 3:** Linear regression analysis of the association between initial IV and IOP in the APAC group.

Variable	Nonadjusted *β* (95% CI)	*P* value	Model I *β* (95% CI)	*P* value	Model II *β* (95% CI)	*P* value
IV	−2.12 (−2.93, −1.32)	<0.001	−1.95 (−2.80, −1.10)	<0.001	−1.85 (−2.77, −0.93)	0.001

Model I: adjusted for age, BMI, and gender. Model II: adjusted for the variables in model I plus hypertension and diabetes.

**Table 4:** Linear regression analysis of the association between IOP and IV in PACS group.

Variable	Nonadjusted *β* (95% CI)	*P* value	Model I *β* (95% CI)	*P* value	Model II *β* (95% CI)	*P* value
IV	0 (−0.15, 0.15)	0.995	−0.02 (−0.19, 0.15)	0.839	−0.02 (−0.20, 0.15)	0.801

Model I: adjusted for age, BMI, and gender. Model II: adjusted for the variables in model I plus hypertension and diabetes.

**Table 5:** Linear regression analysis of correlation between LT and various indicators in all 60 eyes.

Variable	Nonadjusted *β* (95% CI)	*P* value	Model I *β* (95% CI)	*P* value	Model II *β* (95% CI)	*P* value
ACV (mm^3^)	−0.01 (−0.01, 0)	0.009	−0.01 (−0.01, 0)	0.002	−0.01 (−0.01, 0)	0.018
ACA (mm^3^)	−0.05 (−0.07, −0.02)	<0.001	−0.06 (−0.09, −0.04)	<0.001	−0.06 (−0.08, −0.03)	<0.001
ACD (mm)	−0.56 (−0.81, −0.32)	<0.001	−0.62 (−0.85, −0.40)	<0.001	−0.58 (−0.82, −0.34)	<0.001
LV (mm)	0.48 (0.23, 0.73)	<0.001	0.49 (0.26, 0.72)	<0.001	0.48 (0.27, 0.70)	<0.001
Nasal IC	0.77 (0.05, 1.49)	0.041	0.92 (0.22, 1.63)	0.013	1.12 (0.38, 1.86)	0.005
Nasal IT750	−0.76 (−1.32∼−0.21)	0.009	−0.81 (−1.35∼−0.28)	0.004	−0.83 (−1.33, −0.32)	0.002

Model I: adjusted for age, BMI, and gender. Model II: adjusted for the variables in model I plus diagnosis, hypertension, and diabetes.

## Data Availability

The raw data supporting the conclusions of this article will be made available by the authors, without undue reservation.
